# MECHANISMS IN ENDOCRINOLOGY: Diabetic cardiomyopathy: pathophysiology and potential metabolic interventions state of the art review

**DOI:** 10.1530/EJE-17-0724

**Published:** 2018-02-12

**Authors:** Eylem Levelt, Gaurav Gulsin, Stefan Neubauer, Gerry P McCann

**Affiliations:** 1 British Heart Foundation Cardiovascular Research CentreUniversity of Leicester, Glenfield Hospital, Leicester, UK; 2 University of Oxford Centre for Clinical Magnetic Resonance ResearchUniversity of Oxford, Division of Cardiovascular Medicine, Radcliffe Department of Medicine, Oxford, UK; † (E Levelt is now at Multidisciplinary Cardiovascular Research Centre and Biomedical Imaging Science DepartmentLeeds Institute of Cardiovascular and Metabolic Medicine, University of Leeds, Leeds, UK)

## Abstract

Heart failure is a major cause of morbidity and mortality in type 2 diabetes. Type 2 diabetes contributes to the development of heart failure through a variety of mechanisms, including disease-specific myocardial structural, functional and metabolic changes. This review will focus on the contemporary contributions of state of the art non-invasive technologies to our understanding of diabetic cardiomyopathy, including data on cardiac disease phenotype, cardiac energy metabolism and energetic deficiency, ectopic and visceral adiposity, diabetic liver disease, metabolic modulation strategies and cardiovascular outcomes with new classes of glucose-lowering therapies.

## Introduction

Diabetes has reached epidemic proportions and is now among the top 10 causes of death worldwide (1). Type 2 diabetes (T2D) is associated with an increased risk of both heart failure (HF) and cardiovascular mortality even in the absence of coronary artery disease (2, 3). Cardiovascular disease is the leading cause of mortality in patients with diabetes, despite advances in treatment (4, 5). HF is a particularly common complication of diabetes (6, 7, 8), with poor outcomes and five-year survival rates of <25% (5). Poorer glycemic control (hazard ratio (HR) 1.32 per percentage point of HbA1c) is an important predictor of HF development (3).

T2D contributes to the development of HF through a variety of mechanisms, including disease-specific myocardial structural, functional and metabolic changes. The term diabetic cardiomyopathy is applied when cardiac structural and haemodynamic changes are not directly attributable to other confounding factors such as coronary artery disease and hypertension, in patients with diabetes (9). This clinical entity is currently poorly understood, but is clearly of significant clinical importance, given the robust association of diabetes with HF and increased cardiovascular mortality.

## Myocardial structural changes in diabetes

Although the link between HF and diabetes had first been suggested by Leyden as early as 1881 (10), it was not until 1972 when Rubler described the evidence that myocardial damage exists in diabetes independently of other vascular diseases (11). They observed ventricular hypertrophy with diffuse fibrotic strands extending between bundles of muscle fibres and myofibrillar hypertrophy on histopathology in a series of post-mortem studies of four diabetic cases and coined the term ‘diabetic cardiomyopathy’.

In the last 2 decades, there has been an expansion in the armamentarium of non-invasive imaging technologies capable of providing detailed information about the structure of the heart in the health and disease. Patients with diabetes have been extensively phenotyped with a nuanced description of disease burden using these technologies, demonstrating the presence of hypertrophic response of the left ventricle (LV) independently of arterial blood pressure (12). However, the strong association among hypertension, and diabetes (13) is universally accepted, with a significant amount of overlap between the complications of diabetes and hypertension (14); making it difficult to distinguish the impact of diabetes from that of hypertension on the myocardial structural changes reported by many studies.

Several alterations in LV geometry have been demonstrated in patients with diabetes. One study has reported a 1% rise in HbA1c level was associated with a 3.0 g increase in LV mass in elderly subjects (15). Although an increased LV mass is independently associated with diabetes, often this increase was shown to be modest (16, 17). LV concentric remodelling represents the main structural characteristic of diabetic heart disease, precedes the development of clinical HF and was shown to be a strong predictor of adverse cardiovascular events (18). There is less evidence that diabetes itself can cause left ventricular dilatation and eccentric remodelling in the absence of CAD, obesity or hypertension (19). Further, LV concentric remodelling was shown to be more strongly predictive of cardiovascular mortality than eccentric remodelling (18).

Interstitial fibrosis has been implicated in the pathogenesis of LVH and has been identified in the more advanced stages of diabetic cardiomyopathy (11). The role of interstitial fibrosis in the pathogenesis of LVH in stable/early diabetic cardiomyopathy is much less clear, as abnormal myocyte hypertrophy rather than fibrosis appears to predominate in the early stages (20). Cardiovascular magnetic resonance (CMR) imaging native and post-contrast T1 mapping for extracellular volume (ECV) quantification allows for non-invasive quantification of myocardial extra cellular matrix expansion, and it was demonstrated that the ECV correlates closely with collagen proportionate area on histology samples obtained from patients with HF (21). Using this technique, two recent studies demonstrated no significant increase in ECV and native T1 mapping in patients with well-controlled T2D, suggesting the absence of significant extra cellular matrix expansion, even in the presence of LV concentric remodelling and diastolic dysfunction (22, 23). In a larger study of consecutive patients referred for CMR without amyloidosis, investigators showed higher median ECV in patients with diabetes (*n* = 231) than in those without diabetes (*n* = 945) (24). However, in this study, 85% of the patients with diabetes had diagnosed hypertension, which confounds the results.

Describing the myocardial structural changes detected in hypertensive heart disease in detail is beyond the scope of this review article. However, given the significant overlap with the diabetic cardiomyopathy phenotype, in summary hypertension results in increasing arterial stiffness and afterload, leading to remodelling of the myocardium due to cardiomyocyte hypertrophy, fibroblast stimulation and then increased collagen formation (3). In a cohort of well-controlled hypertensive patients, CMR T1 mapping revealed increased diffuse myocardial fibrosis, with small increases in T1 values which were only detected in patients with significant LV hypertrophy (25). Another study has shown concentric LV hypertrophy to be more prevalent than eccentric remodelling in hypertensive patients (26).

## Myocardial functional changes in diabetes

Despite the link with HF on a population level (12), the majority of studies report that diabetes has little or no effect on global LV ejection fraction (LVEF), with the exception of the Strong Heart Study, which has demonstrated the presence of a mild reduction in LVEF (16). However, diabetes traditionally has been linked to diastolic dysfunction mainly based on echocardiography. Consequently, diastolic abnormalities have been suggested as the earliest functional effect of diabetic cardiomyopathy, with reported prevalence rates in asymptomatic, normotensive patients with T2D varying from 15 to as high as 75 per cent (27). The Strong Heart Study demonstrated that the extent and frequency of diastolic dysfunction was directly proportional to the HbA1c level (16).

The combination of pulsed tissue Doppler velocity of the medial mitral annulus (e′) with early passive transmitral inflow velocity (E) has been validated as a reliable index of left ventricular filling pressure. E/e′ ratio has been shown to be a useful prognostic biomarker in diabetic patients. Importantly, abnormality in E/e′ was shown to be associated with insulin resistance (28). From and coworkers in large study of 1760 diabetic patients with a tissue Doppler echocardiographic assessment showed that abnormalities in E/e’ in diabetic patients is associated with the subsequent development of HF and increased mortality independent of hypertension, coronary disease or other echocardiographic parameters (29).

The recent use of relatively less load dependent, sensitive measures of myocardial function with strain imaging by echocardiography and CMR has demonstrated the presence of subtle systolic dysfunction to be frequent as a marker of subclinical heart disease in diabetic patients. Both reduced longitudinal contractility and impaired systolic circumferential strain have been shown in diabetics (30). Although these subclinical abnormalities in contractility are widely considered to be a precursor to the onset of clinical HF in diabetes, prognostic data on the use of strain measures in diabetes is lacking and large longitudinal studies will need to assess this and better define the spectrum of diabetic heart disease.

## Myocardial metabolic changes in diabetes

### Myocardial energy metabolism in diabetes

Maintenance of adequate levels of cardiac high-energy phosphate metabolites, ATP, the energy source for contraction and phosphocreatine (PCr), the major energy storage compound, are of vital importance for normal heart function. Altered myocardial metabolism has been widely considered among the potential mechanisms leading to diabetic heart disease. In the normal heart, 60–90% of ATP synthesis is generated from fatty acids (FA), with a lesser proportion (10–40%) from glucose (31). In diabetes, insulin fails to suppress hormone-sensitive lipase in adipose tissue and very low-density lipoprotein secretion in the liver leading to high circulating FAs. This, in turn activates peroxisome proliferator activated receptor-α (PPARα), which upregulates myocardial FA uptake and metabolism while decreasing glucose transporter 4 (GLUT4) (32, 33). Hence, these systemic metabolic changes in diabetes modify metabolism in the heart, culminating in abnormal cardiac substrate utilisation, impaired cardiac efficiency and decreased energy generation (19, 34, 35, 36, 37). FA regulate glucose metabolism in the heart by activating pathways that lead to the attenuation of insulin signals, thereby inhibiting insulin-mediated glucose transport (38, 39, 40). Due to increased FA availability as a substrate and increased gene expression of FA oxidation enzymes via peroxisome PPARα activation, the β-oxidation increases. This increase in FA availability, and consequently, increased cardiac usage (36, 41, 42, 43, 44, 45, 46), is thought to result in a loss of metabolic flexibility, efficiency between substrate use and ATP production in the diabetic heart (46). The free energy yielded by hydrolysis of ATP is affected by the substrate oxidized (47) and this is lower when excess FA are used compared to glucose (48, 49, 50), resulting in mitochondrial inefficiency and lower ATP yield.

### Myocardial energetic impairment in patients with type 2 diabetes

The relative concentration of PCr to ATP (PCr/ATP) is a sensitive index of the energetic state of the myocardium (31). Phosphorus magnetic resonance spectroscopy (^31^P-MRS) allows non-invasive assessment of the myocardial PCr/ATP. Decreased PCr/ATP is a predictor of mortality (31), linked to contractile dysfunction (51) and is a well-recognized complication of diabetes (30). This pre-existing energetic deficit in diabetic cardiomyopathy is exacerbated by exercise (30). Additionally, exercise PCr/ATP was shown to correlate with impaired myocardial perfusion and oxygenation, suggesting that, in diabetes, coronary microvascular dysfunction exacerbates derangement of cardiac energetics under conditions of increased workload (30).

Although significant correlations between myocardial systolic strain and PCr/ATP were demonstrated (30), the causal role of altered energetics in contractile dysfunction in diabetic hearts remains unclear, and additional research is therefore necessary to delineate the role of myocardial energetics in the development of cardiac dysfunction in patients with T2D.

### Manipulation of substrate utilisation

**Figure 1:**
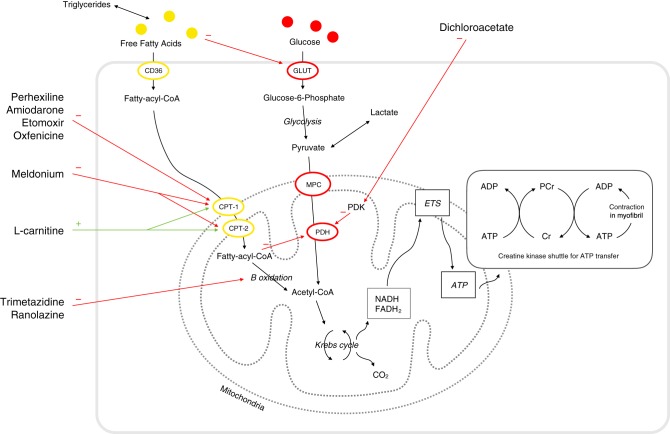
Cardiac energy metabolism and the sites of action of the different metabolic modulators. These range from (A) mitochondrial carnitine shuttle (CPT inhibitors) and (B) β-fatty acid oxidation inhibitors (C) pyruvate dehydrogenase (PDH) activators. In practice, the latter two represent the clinically pertinent therapeutic targets. Group (A) includes perhexiline, etomoxir, oxfenicine and to a lesser extent amiodarone. Group (B) includes trimetazidine and ranolazine. Group (C) includes dichloroacetate. GLUT denotes glucose transporter, PCr phosphocreatine, Cr free creatine.

Epidemiological data have shown an association between glycaemia and incident HF events in patients with or at risk of T2D (52, 53, 54, 55); as a result, major emphasis has been placed on the carbohydrate mechanism. Paradoxically, overall, glucose-lowering drugs or strategies increased the risk of HF compared with standard care (56). There is therefore a need for new and effective alternative therapeutic strategies to reduce the prevalence and incidence of HF in patients with T2D. As such substrate metabolism has become a potential target of pharmacological agents to improve the cardiac function. Myocardial utilization of the glucose and FAs are regulated by substrate availability, competition at the level of the mitochondria and also at the site of cellular entry (57) (Fig. 1). Thus, agents that affect mitochondrial substrate uptake or cellular substrate uptake have been developed. Table 1 includes a list of potential therapeutic strategies to restore the balance of fuel utilisation.

**Table 1:** Potential therapeutic strategies to restore the balance of fuel utilisation in type 2 diabetes.

Strategy/Agent	Effect
Pyruvate dehydrogenase kinase 1–4 inhibition	
Dichloroacetate	Increased flux through PDH Increased Krebs cycle flux Increased oxidative metabolism Pyruvate dehydrogenase activation
CPT-1 inhibition	
Perhexiline, Amiodarone, Etomoxir, Oxfenicine	Decreased fatty acid oxidation Increased glucose oxidation
Long-chain 3-ketoacyl-CoA thiolase inhibitors	
Trimetazidine, Ranolazine	Decreased fatty acid oxidation Increased glucose oxidation
γ-butyrobetaine hydroxylase partial inhibition	
Meldonium	Decreased l-carnitine synthesis Increased glucose oxidation
Carnitine acylcarnitine transferase activation	
l-propionylcarnitine l-carnitine	Increased fatty acid transport across mitochondrial membrane Increased glucose oxidation

Supporting the hypothetical cardiovascular beneficial influences of reduced FA oxidation in animal models of T2D, trimetazidine was shown to ameliorate features of diabetic cardiomyopathy and reverse insulin resistance (58). Trimetazidine is a piperazine derivative with pleiotropic beneficial properties (e.g. anti-ischaemic, antioxidant and even anti-apoptotic roles have been reported) (59). It is licensed as an anti-anginal agent, which selectively inhibits long-chain 3-ketoacyl coenzyme A thiolase (the last enzyme involved in β-oxidation) activity, thereby modifying energy metabolism by partial inhibition of FA oxidation. Although there is a lack of large-scale clinical trials with metabolic modulators, there have been 16 randomised controlled clinical trials of patients with chronic HF suggesting the efficacy of trimetazidine. Trimetazidine was shown to reduce all-cause mortality, improve LVEF, reduce symptoms of HF and plasma B-type natriuretic peptide (BNP) levels (59). While studies reported beneficial effects of trimetazidine on clinical prognosis of diabetic patients with advanced ischaemic heart disease (60), whether or not these beneficial effects can prevent the development of HF in patients with T2D at an early stage has not been explored in clinical studies.

Perhexiline, 2-(2,2-dicyclohexylethyl) piperidine is another metabolic agent reducing FA metabolism through the inhibition of carnitine palmitoyltransferase, the enzyme responsible for mitochondrial uptake of long-chain FA. Perhexiline was also shown to improve LVEF, resting and peak stress myocardial function and skeletal muscle energetics, peak exercise oxygen consumption (VO_2_max) reduce HF symptoms in patients with chronic HF (61), and in a separate study, perhexiline improved myocardial energetics (62). Efficacy of perhexiline was also shown in patients with symptomatic hypertrophic cardiomyopathy (HCM), with improved myocardial energetics, diastolic function and exercise capacity (63). In patients with ischaemic cardiomyopathy, perhexiline was shown to have no effect on wall motion response to dobutamine stress and adverse effect on strain rate compared to placebo (64). Neither of these antianginal metabolic modulators showed any negative inotropic effect to reduce the cardiac workload, consequently, their beneficial cardiovascular effects are considered to be related to inhibition of FA uptake and a metabolic shift towards the use of glucose and lactate (61, 62).

L-carnitine plays a pivotal role in both FA and carbohydrate metabolism. It is responsible for transfer of long-chain FA into the mitochondrial matrix. Meldonium is also an antianginal drug, which partially inhibits *γ*-butyrobetaine hydroxylase, reducing L-carnitine biosynthesis and uptake and consequently leading to a shift away from FA metabolism towards glucose metabolism. In animal models of obesity and impaired glucose tolerance, meldonium reduced plasma insulin concentration and increased cardiac and hepatic PPAR-α activity (65).

There is evidence that increased FA utilisation may ‘paradoxically’ have beneficial effects on cardiovascular health in patients with T2D (66). In the Fenofibrate Intervention and Event Lowering in Diabetes (FIELD) study, PPAR-α agonist fenofibrate treatment was associated with a statistically non-significant trend towards a reduction in the 5-year CVD risk of 14.5 to 13.1%, representing a proportional risk reduction of 11% (adjusted HR 0.89 (95% CI 0–21%), *P* = 0.052; absolute risk reduction 1.4%). These contradictory outcomes make it even more pertinent to delineate the precise metabolic changes that occur in patients with T2D. Rather than representing a paradox, this may indicate that it is the lack of metabolic flexibility, rather than specific substrate preference that predisposes the diabetic heart to injury (67). Further, PPAR-α activation may be beneficial with two significant advantages: (i) providing continued support to the muscle’s metabolic needs and (ii) avoiding accumulation of lipid byproducts that could be harmful to the cardiomyocyte (68). Additionally, pleiotropic benefits of these agents may be responsible for these beneficial effects (67).

### Myocardial steatosis in patients with type 2 diabetes

Excess myocyte accumulation of lipids has emerged as an important contributor to the development of diabetic cardiomyopathy, particularly concentric LV remodelling (23). The discordance between the rates of FA availability and/or uptake with that of FA oxidation results in increased intracellular long-chain fatty acyl-CoA concentrations (19). Since cardiomyocytes are not specialised to store lipid, this finding suggests a deleterious effect and cellular lipid overloading underlies the concept of ‘lipotoxicity’ as a potential mechanism for impaired cardiac function (32). The excess long-chain fatty acyl-CoA is then diverted towards non-oxidative processes with the production of lipotoxic intermediates such as ceramide and diacyl-glycerol (32). These have been shown to activate signalling pathways affecting ATP production, insulin sensitivity, myo-cellular contractility and apoptosis (32, 69). Increased FA levels stimulate cardiac PPAR-α, resulting in upregulation of the lipid metabolising pathway, and PPAR-α-overexpressing mice show a phenotype similar to diabetes (32). This provides another potential mechanistic link between cardiac steatosis, lipotoxicity and concentric LV remodelling in diseases of upregulated FA metabolism such as diabetes.

Using proton (^1^H)-MRS, myocardial triglyceride content has been shown to be increased by 1.5- to 2.3-fold in T2D (23, 70). Importantly, myocardial steatosis has been shown to be modifiable (71, 72). Successful reduction of myocardial steatosis with GLP-1 agonists (71) and mineralocorticoid receptor blockers (72) have both been shown to reverse concentric LV remodelling. However, larger studies targeting myocardial lipid accumulation are needed to confirm these observations.

## Ectopic and visceral adiposity and insulin resistance in patients with type 2 diabetes

**Figure 2:**
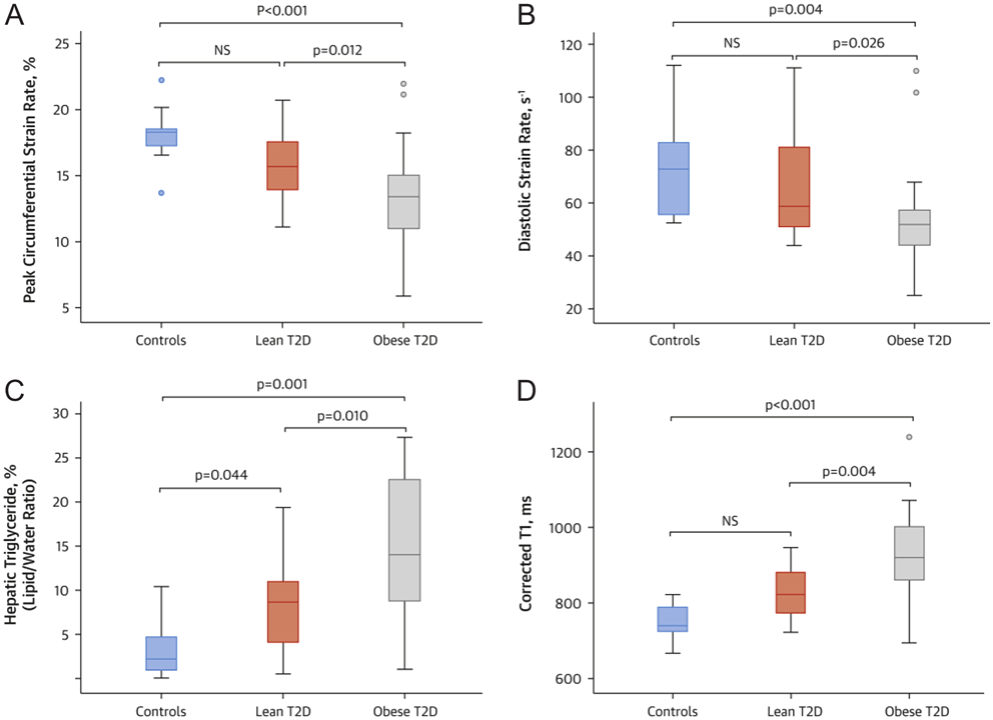
Differences in cardiac function, hepatic steatosis, and hepatic cT1 among the study cohorts. (A) Peak circumferential systolic strain; (B) diastolic strain rate; (C) hepatic triglyceride content (%) and (D) hepatic corrected T1 map (ms). The dots indicate values outside the interquartile range. Reproduced with permission from Levelt *et al*. (83). Copyright© The American College of Cardiology.

Accumulating evidence suggests that: (i) the distribution of excess fat is an important determinant of cardiovascular risk; (ii) ectopic and visceral adiposity confer a much higher risk than subcutaneous adiposity (73, 74) and (iii) abnormal distribution of excess fat may also play a role in the pathogenesis of cardiomyopathy process associated with diabetes and obesity (75). Computed tomography (CT), MRI, ultrasonography and ^1^H-MRS have all been used to quantify adipose tissue amount or lipid content within an organ and to examine the association of various fat depots with both systemic and local manifestations of disease (70, 76, 77, 78, 79, 80). Recently, using these techniques, it was demonstrated that, irrespective of body mass index, diabetes is related to significant abnormalities in cardiac function, energetics and cardiac and hepatic steatosis (81). However, obese patients with T2D showed a greater propensity for ectopic fat deposition that is associated with cardiac contractile dysfunction and fibroinflammatory liver disease than lean T2D patients (81) (Fig. 2).

Epicardial adipose tissue (EAT), which is a form of visceral fat, has no anatomical barriers with the myocardium, and, by secreting proinflammatory adipokines and cytokines through paracrine/autocrine signalling pathways, EAT may play a significant role in diabetic heart disease. Supporting this theory, an inverse correlation of EAT volumes with cardiac systolic strain was demonstrated (81). Similarly, excess liver fat, which is a form of ectopic fat, has been shown to be accompanied by cardiac structural and functional changes (82).

Ectopic and visceral adiposity or ‘acquired lipodystrophy’ is linked to insulin resistance and diabetes (83). Multiple studies support the concept that insulin resistance is prompted, and sustained by, dysregulated fat tissue (84, 85, 86). It is possible that the insulin resistance may be responsible for the increased cardiovascular risk that is linked to ectopic and visceral adiposity. Additionally, there is evidence for a strong association between insulin resistance and non-ischaemic HF (87). There are many molecular mechanisms that may contribute to the association between insulin resistance and non-ischaemic cardiomyopathy (87). These include metabolic inefficiency (19), impaired vascular function (88), inflammation, mitogenic actions of insulin on myocardium leading to changes of left ventricular geometry (89). However, there are differing opinions whether this relationship is of protective or pathological nature (90, 91, 92). Although it has been demonstrated that insulin resistance and ectopic adiposity are associated with an even greater cardiovascular risk (93, 94), Nolan and coworkers recently argued that insulin resistance protects critical tissues, such as the heart, from nutrient-induced damage (92). It has been proposed that insulin resistance is an antioxidant defence mechanism (90). Consequently, lately, there has been something of a paradigm shift in the consensus regarding the nature of the role of insulin resistance in diabetes associated cardiovascular risk, when the traditional thinking had regarded insulin resistance as a primary etiological factor in the development of non-ischaemic HF. This is based on the evidence that impairment of mitochondrial oxidative capacity, which follows increased reactive oxygen species (ROS) production in muscles of diet-induced diabetic mice (95) and inhibition of mitochondrial ROS production reverses insulin resistance (96, 97). This novel perspective might offer answers to why some previous research therapeutically targeting impaired insulin sensitivity resulted in deleterious effects such as PPARs, including the development of HF in patients with diabetes (90, 98).

## Novel glucose-lowering therapies and improved cardiovascular outcomes

Recently new classes of glucose-lowering therapies, such as glucagon-like peptide-1 (GLP-1) analogues (99) and inhibitors of sodium–glucose cotransporter 2 (7) have shown exciting results with improved glycaemic control as well as reduced cardiovascular mortality in patients with T2D. However, these recent trials were designed to assess the specific effects of these novel drugs on clinical outcomes, and therefore, the mechanisms behind the observed cardiovascular benefits are speculative. It would be interesting to see if the potential beneficial effects of these novel therapies on cardiovascular health will be reflected by the changes measured with non-invasive imaging techniques.

The biologic action of GLP-1 is focused on the intake, absorption, retention and disposal of energy-rich substrates (100). In normal physiology, endogenous GLP-1 is implicated in the control of appetite and satiety, not surprisingly therefore GLP-1 is currently under intensive investigation as a potential primary mediator of beneficial metabolic effects after bariatric surgery, with its eating-inhibitory, antiobesity and antidiabetes effects (101). The principal determinants of the levels of active plasma GLP-1 include enzymatic inactivation by dipeptidyl peptidase 4 (DPP-4) and neutral endopeptidase and renal clearance (102).

The incretin-based drugs include dipeptidyl peptidase 4 (DPP-4) inhibitors and GLP-1 analogues. The GLP-1 analogues exert their effect via the incretin system, specifically targeting the receptor for the incretin hormone GLP-1, which is partly responsible for augmenting glucose-dependent insulin secretion in response to nutrient intake (the ‘incretin effect’). The predominant actions of exogenously administered GLP-1 regulate blood glucose via inhibition of appetite, glucagon secretion and gastric emptying and stimulation of insulin secretion (103).

GLP-1 receptors are also expressed in the heart, and administration of GLP-1 improves cardiovascular function in the setting of experimental cardiac injury (104). The actions of GLP-1 on the heart may be directly through generation of cAMP in cardiomyocytes and/or indirectly by improvement of the metabolic environment through control of blood glucose, insulin and Fas (105). The Liraglutide Effect and Action in Diabetes: Evaluation of Cardiovascular Outcome Results (LEADER) trial showed that death from cardiovascular causes occurred in fewer patients in the GLP-1 analogue liraglutide group compared to the placebo group in patients with T2D and high cardiovascular risk. Similarly, in high-risk T2D patients, the rate of cardiovascular death, nonfatal myocardial infarction or nonfatal stroke was significantly lower for semaglutide than for placebo (106). Exenatide is an exendin-4-based GLP-1 receptor agonist which is a once-weekly, injectable, extended-release formulation drug. The Exenatide Study of Cardiovascular Event Lowering (EXSCEL) assessed the long-term cardiovascular safety and efficacy of exenatide, in patients with T2D who had a wide range of cardiovascular risk (107). The results of this study showed that exenatide was non-inferior to placebo with respect to cardiovascular safety, but it was not superior to placebo with respect to efficacy. The risk of death from any cause was 6.9% in the exenatide group and 7.9% in the placebo group (hazard ratio, 0.86; 95% CI, 0.77–0.97); this difference was not statistically significant. Furthermore, the Functional Impact of GLP-1 for Heart Failure Treatment (FIGHT) study which was a multicentre, double-blind, placebo-controlled randomized clinical trial of patients with established HF and reduced LVEF has demonstrated that liraglutide does not improve post-hospitalization clinical stability in patients with advanced HF and reduced LVEF despite prior studies indicating that GLP-1 therapy might ameliorate mechanisms of myocardial insulin resistance reported in patients with severe cardiomyopathies (108). No favourable effects of liraglutide on secondary end points based on echocardiographic measures, 6-minute walk distance or quality of life scores were shown. The negative outcome in this study was speculated to be potentially associated with the promotion of glucose-dependent insulin secretion with GLP1 agonists. This is also providing extra support for the argument put forward recently by Nolan and coworkers that insulin resistance protects critical tissues, such as the heart, from nutrient-induced damage (92) that enhancing endogenous insulin secretion is disadvantageous in the setting of HF, and myocardial insulin resistance in HF models might be an adaptive mechanism in patients with advanced HF.

Inhibitors of DPP-4 reduce the breakdown of endogenous GLP-1. Unlike with GLP-1 analogues, there has been considerable speculation about the potential beneficial effects of DPP-4 inhibitors on the cardiovascular system. The results of multicentre observational studies of DPP4 inhibitors showed no beneficial results on their effect on HF admissions: (i) SAVOR–TIMI 53 trial (109) showed 27% increase in the risk of hospitalization for HF in patients with T2D assigned to saxagliptin compared to those who received placebo, and the drug did not provide any cardioprotective benefit; (ii) cardiovascular outcomes studies EXAMINE trial (110) of alogliptin vs standard care and TECOS trial (111) of sitagliptin both showed no increase in the overall risk of hospitalization for HF among patients randomly assigned to alogliptin and sitagliptin, vs standard care respectively. Meta-analysis of several large cohorts of patients with T2D, the use of incretin-based drugs, as compared with combinations of oral antidiabetic drugs, was not associated with an increased risk of hospitalization for HF (112).

The incretin-based drugs were also shown to reduce the occurrence and degree of hepatic steatosis independent of their action on body weight in an experimental study (113) and also in a small clinical phase 2 study in overweight patients with nonalcoholic steatohepatitis (114).

Finally, inhibitors of sodium–glucose cotransporter 2 reduce rates of hyperglycemia in patients with T2D by decreasing renal glucose reabsorption, thereby increasing urinary glucose excretion (115). EMPA-REG OUTCOME trial showed that patients with T2D at high risk for cardiovascular events who received empagliflozin, a selective inhibitor of sodium–glucose cotransporter 2, had significantly lower rates of the primary composite cardiovascular outcome and of death from any cause than did those in the placebo group when the study drugs were added to standard care with almost immediate beneficial effect despite a modest improvement in glycaemic control, with approximately 0.4% reduction in glycated haemoglobin of over 94 weeks (7). Canagliflozin is another SGLT2 inhibitor. The Canagliflozin Cardiovascular Assessment Study (CANVAS) Program, comprising two sister trials, was designed to assess the cardiovascular safety and efficacy of canagliflozin. The trial program showed that T2D patients with high risk of cardiovascular disease treated with canagliflozin had a significantly lower risk of death from cardiovascular causes, nonfatal myocardial infarction or nonfatal stroke than those who received placebo, but they were at a greater risk of amputation (116). The CVD-REAL Study was a retrospective registry study designed to evaluate the association of outcomes of hospitalization for HF and all-cause death in patients with T2D treated with SGLT-2 inhibitors vs other glucose-lowering drugs. Consistent with the EMPA-REG OUTCOME, CVD-REAL Study showed treatment with SGLT-2 inhibitors was associated with 39% relative risk reduction in HF hospitalization, a 51% reduction in all-cause mortality. These beneficial effects, particularly relevant to HF admissions, appeared to be class related. Importantly, overwhelming majority (87%) of patients included in the study had no established CVD, suggesting that lower risk patients may derive similar benefits with SGLT-2 inhibitors, as those with higher risk. However, this study similar to others did not address the mechanisms linking use of SGLT-2 inhibitors and associated cardiovascular benefits (117).

As a result, the reasons for the beneficial cardiovascular effects are not yet clear, however recently suggested theories include: (i) SGLT2 inhibitor induced plasma volume contraction (5% increase in haematocrit in conjunction with a 35% relative risk reduction in hospital admission for HF on empaliflozin arm) (118); (ii) Restoring cellular energy homeostasis by activation of AMPK (119, 120); (iii) SGLT2 inhibitor induced mild ketosis (121, 122). This may improve myocardial/renal metabolic efficiency and function, given that the ketone body oxidation yields more ATP per oxygen consumption than palmitate, therefore, being more ‘energy-efficient’ (49).

## Diabetic cardiomyopathy in type 1 diabetes

Relatively little research has taken place comparing the underlying mechanisms and clinical features of diabetic cardiomyopathy in type 1 vs type 2 diabetes. Although high prevalence of subclinical myocardial dysfunction has been reported in the early stage of type 1 diabetes (T1D), clinical presentations of HF is relatively rare in this type of diabetes compared to T2D (123). In a longitudinal observational study of a relatively large cohort of T1D patients without a previous history of heart disease only 17 patients out of 462 (3.7%) were shown to develop HF during a 12-year follow-up period (124). Those patients who developed HF were reported to be older with a longer duration of diabetes (35 ± 9 years), and had higher blood pressure, and higher prevalence of albuminuria and retinopathy compared to those without HF.

Similar to T2D (125), cardiomyocyte hypertrophy has been reported for different animal models of T1D (126), however significant reduction in the cardiomyocyte cross sectional area was also observed in a model of T1D (127). Fewer studies of T1D have shown an increase in LV mass compared to T2D. This may be due to the younger age and lower incidence of hypertension in T1D patients investigated in most studies. Significant LV dysfunction has also been detected by tissue Doppler and speckle tracking echocardiography techniques in T1D patients (128). Myocardial metabolic remodelling studies have been scarce in T1D. A single study demonstrated a significant reduction in myocardial energetics at rest independently of myocardial perfusion reserve changes, similarly to T2D (129). To our knowledge no studies to date have evaluated the role of myocardial steatosis in T1D.

## Conclusions

Science has progressed significantly in its understanding of disease mechanisms in type 2 diabetes, and significant advances have been made in characterizing the metabolic phenotype in the diabetic heart and in defining the relationship among the myocardial metabolic remodelling, structural and functional changes. However, the fundamental question of whether or not a primary alteration in substrate utilisation in diabetes is responsible for cardiac dysfunction remains uncertain. The ability to manipulate cardiac metabolism is a promising therapeutic target which may shed light on this question. The mechanisms behind the observed cardiovascular mortality benefits of new classes of glucose-lowering therapies also remain to be shown. In search of treatment and prevention of diabetes-associated HF, the road ahead still appears long, but promises significant advances.

## Declaration of interest

All authors have read and understood *European Journal of Endocrinology* policy on declaration of interests and declare that we have no competing interests.

## Funding

This work was supported by the Wellcome Trust (grant number 207726/Z/17/Z).

## Authors contribution statement

All authors made appropriate contributions according to the ICMJE guidance, and as such have read and approved the final manuscript. All authors take public responsibility for appropriate portions of the manuscript content; and agree to be accountable in ensuring that questions related to the accuracy or integrity of the work are appropriately investigated and resolved. E L, G S, S N and G M each contributed to drafting of manuscript and revisions.
